# Contact Sensitizers Induce Skin Inflammation via ROS Production and Hyaluronic Acid Degradation

**DOI:** 10.1371/journal.pone.0041340

**Published:** 2012-07-25

**Authors:** Philipp R. Esser, Ute Wölfle, Christoph Dürr, Friederike D. von Loewenich, Christoph M. Schempp, Marina A. Freudenberg, Thilo Jakob, Stefan F. Martin

**Affiliations:** 1 Allergy Research Group, Medical Center, University Freiburg, Freiburg, Germany; 2 Department of Dermatology, Competence Center Skintegral, Medical Center, University Freiburg, Freiburg, Germany; 3 Department of Haematology and Oncology, Medical Center, University Freiburg, Freiburg, Germany; 4 Institute of Medical Microbiology and Hygiene, Medical Center, University Freiburg, Freiburg, Germany; 5 Max-Planck-Institute for Immunobiology and Epigenetics, Freiburg, Germany; University of Patras, Greece

## Abstract

**Background:**

Allergic contact dermatitis (ACD) represents a severe health problem with increasing worldwide prevalence. It is a T cell-mediated skin disease induced by protein-reactive organic and inorganic chemicals. A key feature of contact allergens is their ability to trigger an innate immune response that leads to skin inflammation. Previous evidence from the mouse contact hypersensitivity (CHS) model suggests a role for endogenous activators of innate immune signaling. Here, we analyzed the role of contact sensitizer induced ROS production and concomitant changes in hyaluronic acid metabolism on CHS responses.

**Methodology/Principal Findings:**

We analyzed *in vitro* and *in vivo* ROS production using fluorescent ROS detection reagents. HA fragmentation was determined by gel electrophoresis. The influence of blocking ROS production and HA degradation by antioxidants, hyaluronidase-inhibitor or p38 MAPK inhibitor was analyzed in the murine CHS model. Here, we demonstrate that organic contact sensitizers induce production of reactive oxygen species (ROS) and a concomitant breakdown of the extracellular matrix (ECM) component hyaluronic acid (HA) to pro-inflammatory low molecular weight fragments in the skin. Importantly, inhibition of either ROS-mediated or enzymatic HA breakdown prevents sensitization as well as elicitation of CHS.

**Conclusions/Significance:**

These data identify an indirect mechanism of contact sensitizer induced innate inflammatory signaling involving the breakdown of the ECM and generation of endogenous danger signals. Our findings suggest a beneficial role for anti-oxidants and hyaluronidase inhibitors in prevention and treatment of ACD.

## Introduction

Allergic contact dermatitis (ACD) is a T cell-mediated delayed type hypersensitivity reaction, which is induced by protein-reactive organic chemicals or metal ions. In mice, the contact hypersensitivity (CHS) model mimics the processes occurring in human ACD. The first skin contact with sensitizing allergens results in activation and migration of allergen-bearing skin DCs to the skin-draining lymph nodes where they complete maturation and present the antigen to allergen specific naive T cells. Subsequently, in a second phase, re-exposure to the same sensitizer results in the recruitment of effector T cells to the inflamed skin and their cytotoxic action on skin cells [1], [2]. The adaptive immune response in ACD is elicited mainly by activation and expansion of cytotoxic CD8+ Tc1 or CD4+ Th1 cells and Tc17/Th17 cells in a multi-step process [3], [4], [5]. In murine CHS the main effector cells are cytotoxic Tc1 cells.

**Figure 1:**
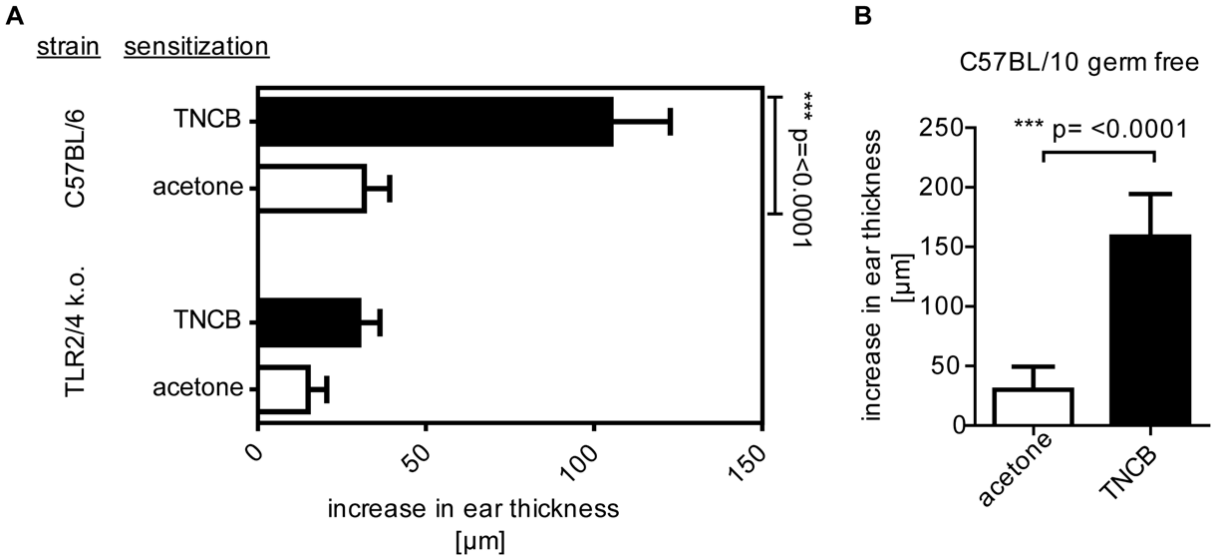
TLR2/4 knockout mice are unable to mount CHS responses to TNCB. (A) WT or TLR2/4 double knock out mice were sensitized with TNCB (3%) or acetone as solvent control and challenged with TNCB (1%) 5 days later. Data show mean increase in ear thickness +/− SD with n = 3 mice/group. One of two independent experiments is shown. (B) Germ-free C57BL/10 mice were treated as in A. Data show mean increase in ear thickness +/− SD of 6 (acetone) or 7 (TNCB) mice. One experiment was performed.

A crucial step for efficient priming of naïve T cells is the induction of a maturation process in DCs. However, in contrast to pathogen induced TLR triggering and subsequent activation of the MyD88 and TRIF dependent signaling pathways by invading pathogens [6], DC activation caused by contact sensitizers is incomplete. The *in vitro* exposure of DCs to 2,4,6-trinitrochlorobenzene (TNCB) leads to the up-regulation of co-stimulatory molecules, but fails to induce a cytokine response [7]. In this case, for full activation of DCs a secondary signal is necessary that is derived from the tissue microenvironment [8], [9], [10], [11], [12]. This signal might be provided by endogenous ligands activating pattern recognition receptors (PRRs). Indeed, our observation that double deficient mice lacking expression of functional IL12Rβ2/TLR4, IL12Rββ2/TLR2 or TLR2/TLR4 are resistant to CHS while expression of the above combination of receptors on murine DCs only is sufficient for the induction of CHS, strongly suggested a role for endogenous TLR2 and TLR4 ligands [7]. Several endogenous molecules, so-called damage associated molecular patterns (DAMPs), have been suggested to elicit immune-stimulatory effects - analogous to microbial pathogen associated molecular patterns (PAMPs) - by triggering TLR or NOD like receptor (NLR) signaling [13], [14]. Among these endogenous molecules are heat shock proteins, uric acid, ATP and ECM components such as biglycan and low MW fragments of hyaluronic acid (HA) [15], [16], [17], [18].

HA is a negatively charged glucosaminoglycan ubiquitously distributed in the ECM [19] and is primarily produced by dermal fibroblasts and epidermal keratinocytes and to a lesser extent by other cell types like smooth muscle cells [20]. Regarding its immune-modulatory effects, the size of HA plays an important role. High MW HA (<1×10^6^ kDa) is anti-angiogenic, anti-inflammatory and immunosuppressive [21], [22], [23]. In contrast, breakdown products occurring in the range from 1.2 to 500 kDa that are generated during inflammation or tissue damage induce pro-inflammatory innate immune responses [24] presumably via TLR2 and/or TLR4 in immune cells like macrophages or DCs [10], [25] and play a pro-inflammatory role in lung inflammation [8], [26]. HA fragments can be generated enzymatically by a group of hyaluronidases, and non-enzymatically by ROS, especially at sites of inflammation, tissue injury and tumorigenesis [27].

The fact that sensitization to the contact sensitizer TNCB was significantly reduced in germ-free mice pre-treated with an inhibitor of HA function (Pep-1) underlined the role of HA in the induction of skin inflammation by contact sensitizers [7].

**Figure 2:**
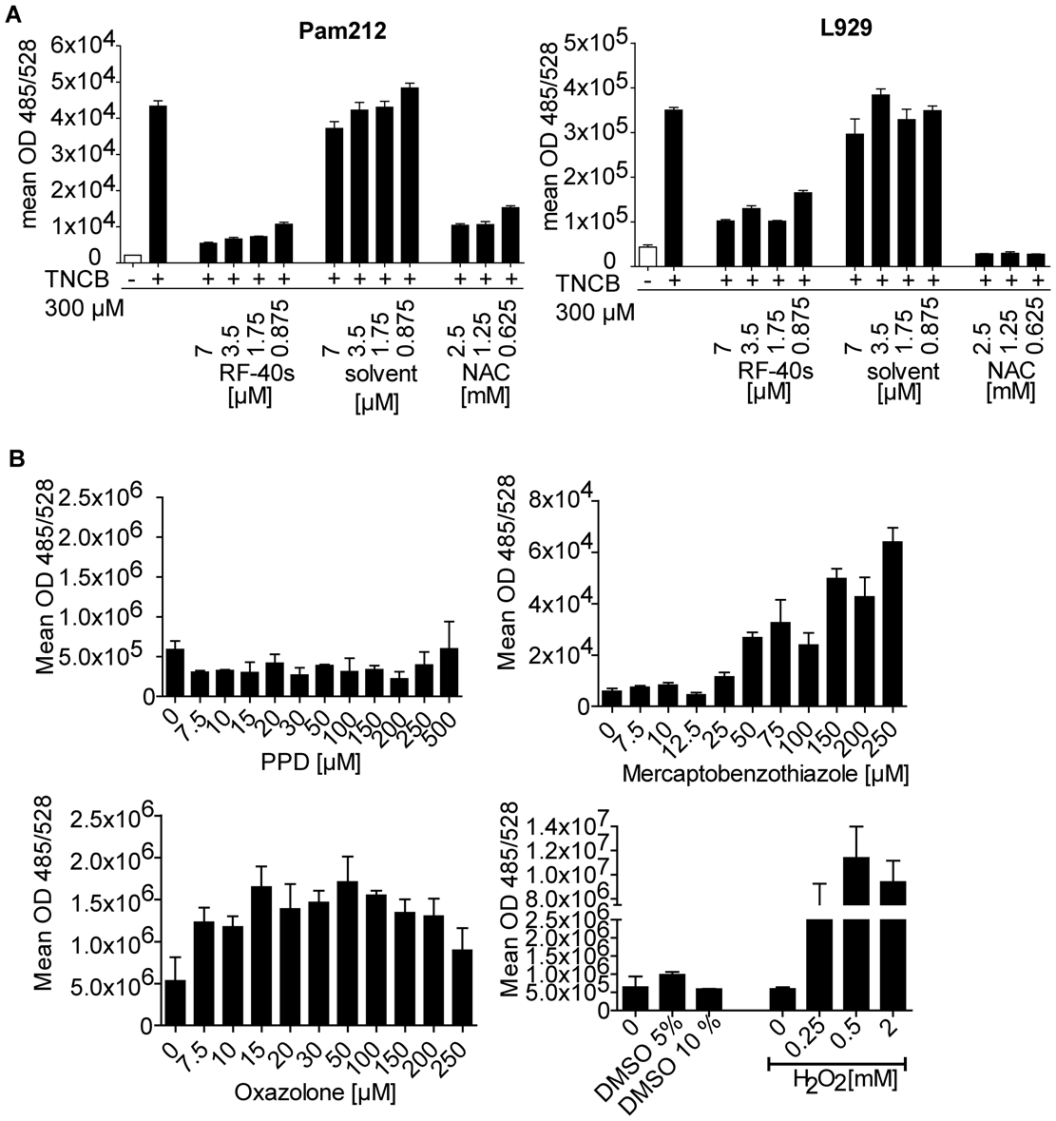
Contact sensitizers induce ROS production in murine Pam212 and L929 cell lines *in vitro.* (A) Pam212 or L929 cells were incubated with CM-H_2_DCFDA (5 µM) for 15 min before addition of RF-40s, solvent or NAC. 15 min later TNCB (300 µM) was added. OD485/528 was measured every 10 min for 1 h. Results show the calculated mean OD +/− SD of triplicate wells of all time points analyzed. One representative of three independent experiments is shown. (B) Mean fluorescence was calculated comprising all time points after incubation of Pam212 cells with CM-H_2_DCFDA and treatment with different concentrations of the pro-hapten paraphenylenediamine (PPD), the extreme sensitizer oxazolone or the moderate sensitizer mercaptobenzothiazole (MBT) as in (A). DMSO and H_2_O_2_ served as solvent and positive control, respectively.

In the present study, we further investigated the role and metabolism of HA as a putative endogenous activator of innate immune signaling necessary to trigger full activation of DCs *in vivo.* We suggest a new mechanism for the generation of a pro-inflammatory milieu by organic contact sensitizers. In contrast to the direct human TLR4 activation by the metal ion nickel [28], organic sensitizers such as TNCB induce ECM degradation thereby providing endogenous activators of the innate immune response. Understanding the underlying molecular mechanisms of this xenoinflammation which is a crucial prerequisite for the development of CHS responses [13], [14] is essential for the development of reliable *in vitro* test systems for the identification of chemicals with skin sensitizing potential [29]. In conclusion, interference with contact sensitizer induced modulation of HA metabolism might help to prevent the innate inflammatory response that is instrumental both for the sensitization and elicitation phase and should, therefore, result in new therapies for ACD.

**Figure 3:**
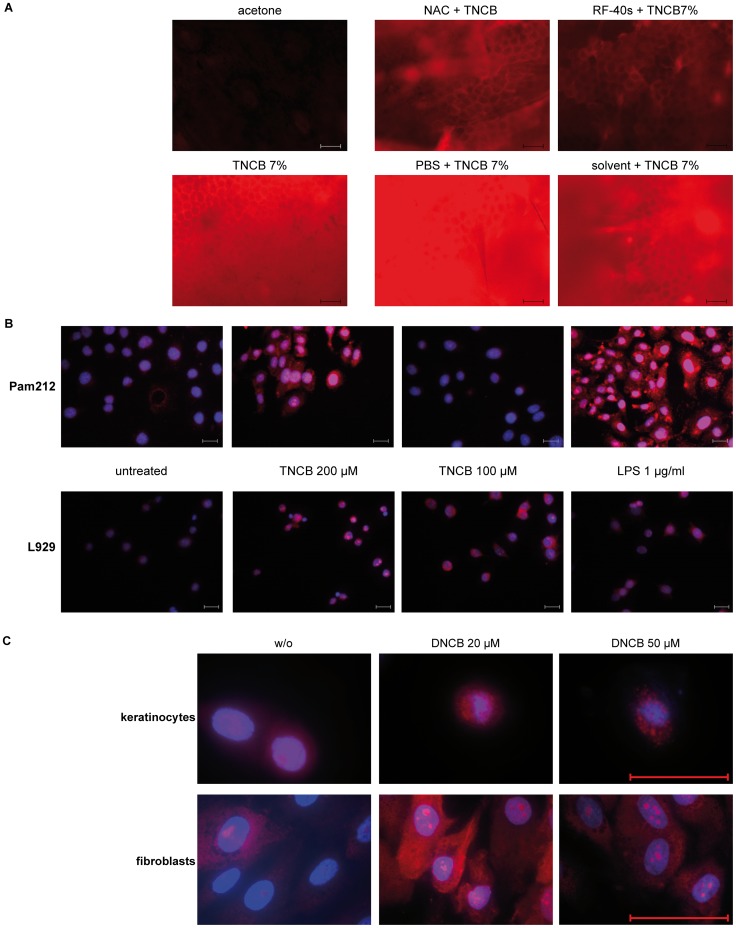
Contact sensitizers induce ROS production *in vivo* and in mitochondria *in vitro.* (A) Mice were pre-treated by topical application of antioxidants (NAC 5 mM or PBS as solvent, RF-40s 5.24 mM or solvent) on the ears. NAC/PBS was applied 1 h before and RF-40s/solvent 15 min before induction of ROS production by topical application of TNCB (7%). Acetone treatment served as solvent control for TNCB. 15 min later, ears were taken after euthanasia and incubated *ex vivo* with DHE (5 mM) in DMSO for 30 min before analysis of ROS production by fluorescence microscopy. Fluorescence was set for minimal background staining with the acetone control to optimize visualization of differences in ROS production in the other samples. Same acquisition times were used for all samples of one experiment. Results shown are representative of three independent experiments. Magnification  = 200×, scale bar  = 50 µm. (B) Pam212 or L929 cells were incubated with TNCB, LPS or left untreated for 1 h before addition of MitoSOX™. ROS production was observed by red/orange fluorescence of MitoSOX™ by fluorescence microscopy. Nuclei were visualized by DAPI staining (blue). Pictures shown are representative of three independent experiments. Magnification  = 400×, scale bar  = 20 µm. (C) Primary human fibroblasts or keratinocytes were incubated with DNCB or left untreated for 1 h before addition of MitoSOX™. ROS production was observed by red/orange fluorescence of MitoSOX™ by fluorescence microscopy. Pictures shown are representative of three independent experiments. Magnification  = 1000×, scale bar  = 50 µm.

## Results

### CHS Responses Depend on TLR2 and TLR4 Signaling but do not Require Exogenous TLR Ligands

C57BL/10 mice lacking TLR2 and TLR4 are resistant to TNCB-induced CHS responses (Figure 1A) and, moreover, CHS responses to TNCB (Figure 1B) and oxazolone (data not shown) are successfully induced in germ-free TLR2- and TLR4-competent C57BL/10 ScSn wildtype mice. These data confirm our previous findings [7] and suggest strongly that endogenous activators of innate signaling such as low MW HA fragments are required in the sensitization and elicitation phase of CHS. Based on our previous demonstration of a role of HA in CHS we focused on HA and its metabolism [7].

**Figure 4:**
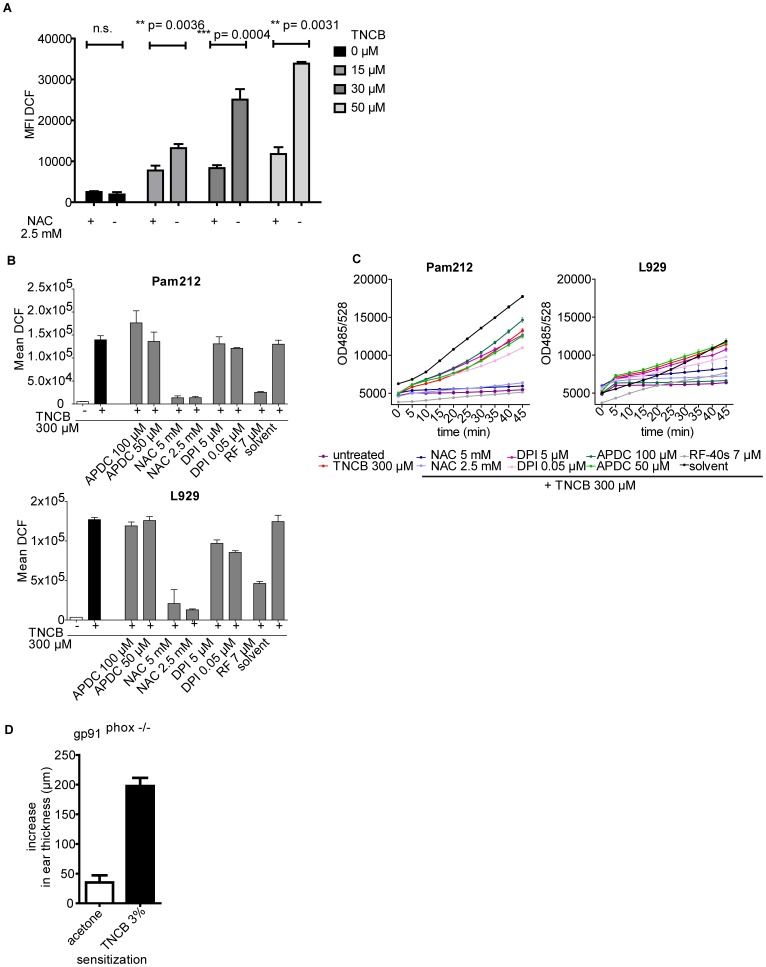
Source and kinetics of TNCB induced ROS production *in vitro.* (A) DCF fluorescence after incubation of BMDC with different concentrations of TNCB for 2 h either with or without NAC treatment was analyzed by flow cytometry. Data show mean fluorescence intensity of DCF +/− SD of triplicate stimulations. One of two independent experiments is shown. (B) Pam212 or L929 cells were left untreated (−) or stimulated with TNCB (300 µM) after pre-treatment with antioxidants NAC or RF-40s for 1 h. In addition, the influence of the mitochondria specific antioxidant APDC and the NADPH oxidase specific antioxidant DPI was analyzed under the same conditions. DCF fluorescence was analyzed 5, 10, 15, 20, 25, 30, 35, 40 and 45 min after TNCB addition. Data show mean fluorescence as calculated comprising all timepoints (B) or kinetics of fluorescence (C) +/− SD of quadruplicate wells from one representative experiment out of three. (D) gp91^phox^ −/− mice were sensitized with TNCB (3%) or mock treated with acetone. 5 days later, mice were challenged with TNCB (1%) and increase in ear thickness was measured 24 h later. Data shows mean increase in ear thickness +/− SD of n = 3 mice/group. One representative of three independent experiments is shown.

### Contact Sensitizers Induce ROS Production *in vitro* and *in vivo*

Pro-inflammatory low MW fragments of HA can be produced by oxidative degradation of high MW HA by ROS [30], [31]. Over-expression of extracellular SOD inhibits this degradation and prevents experimental lung inflammation [32] and CHS in mice [33]. Therefore, we analyzed the ability of the strong contact sensitizer TNCB to induce ROS production in the murine keratinocyte cell line Pam212 and in the fibroblast cell line L929 *in vitro* (Figure 2A). We observed ROS production that was efficiently blocked by the plant derived antioxidant RF-40s [34] as well as by the antioxidant N-acetylcysteine (NAC) (Figure 2A). Furthermore, dose-dependent ROS production was observed in Pam212 cells also after treatment with the extreme sensitizer oxazolone and the moderate sensitizer mercaptobenzothiazole (MBT) [35], [36], but not with the non-sensitizing pro-hapten para-phenylene diamine (PPD) (Figure 2B). H_2_0_2_ treatment served as positive control (Figure 2B).

**Figure 5:**
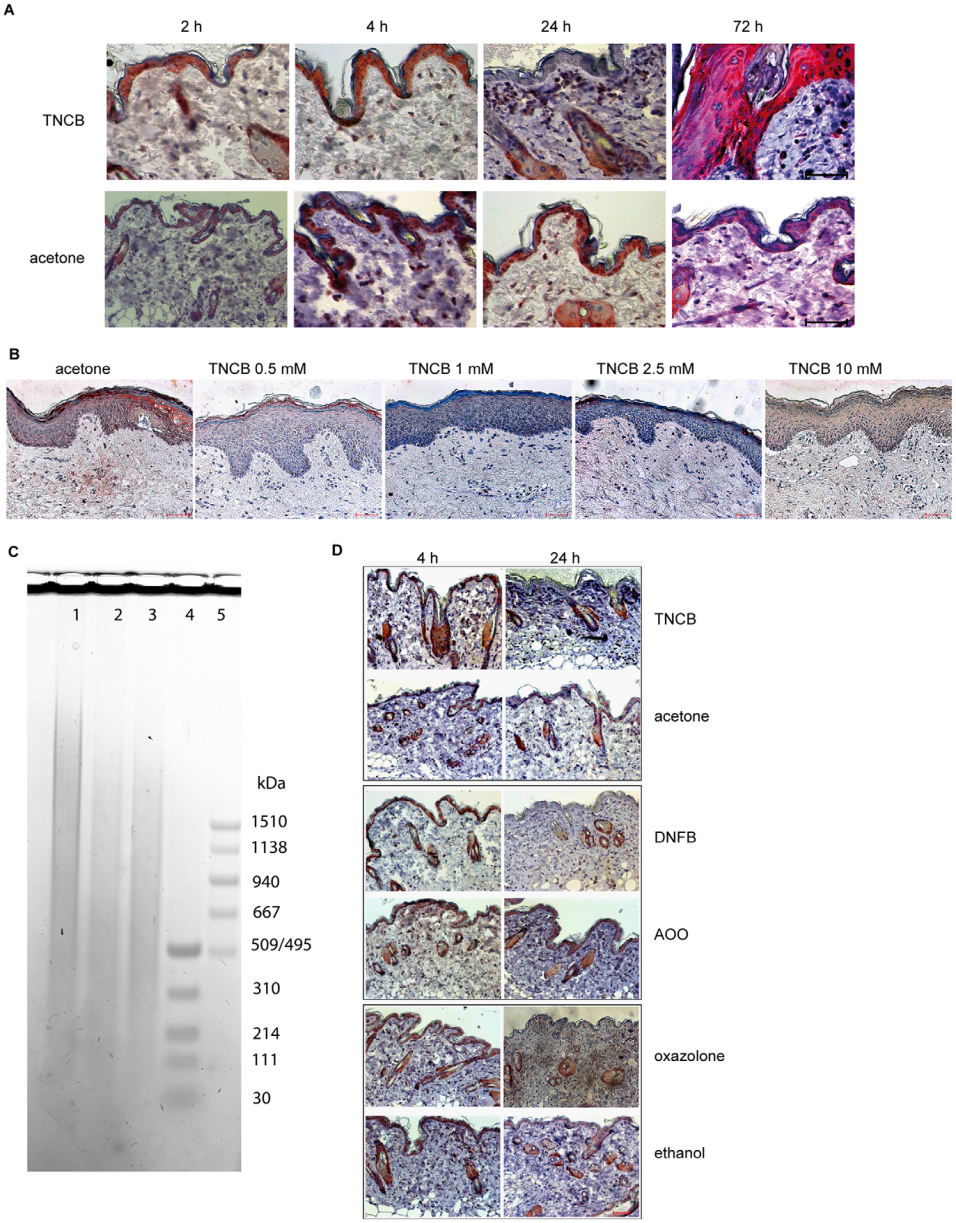
Contact sensitizers induce HA degradation *in vivo.* (A) Staining of HA in TNCB or acetone treated murine skin. Biopsy samples from murine abdominal skin were fixed as described and paraffin sections (3 µm) were stained with bHABP with subsequent AEC (3-Amino-9-ethylcarbazole) staining and haematoxylin counterstaining. HA is stained brown/red and cell nuclei in blue. Pictures are representative of three independent experiments with three mice each. Magnification  = 400×, scale bar  = 50 µm. (B) Staining of HA in TNCB treated human skin. Biopsy samples were treated with indicated concentrations of TNCB for 24 h and afterwards treated like the murine samples above. Pictures are representative of samples from three independent donors. Magnification  = 200×, scale bar  = 50 µm. (C) Abdominal skin was excised either untreated (lane 1), or 4 h (lane 2) or 24 h (lane 3) after application of TNCB (3%). Samples (8 mm diameter) were digested and the molecular weight of HA of the samples was determined by agarose gel electrophoresis. In lane 4 and 5 molecular weight markers (5 µl/lane of HiLadder or LoLadder) were loaded. The picture shows one representative gel out of three. (D) Abdominal mouse skin was treated with sensitizers (TNCB, DNFB or oxazolone) and HA staining was performed 4 or 24 h later. Respective solvent controls (acetone, acetone/olive oil (AOO), ethanol) are shown below. Biopsy samples from murine abdominal skin were fixed as described and paraffin sections (3 µm) were stained with bHABP with subsequent AEC (3-Amino-9-ethylcarbazole) staining and haematoxylin counterstaining. HA is stained brown/red and cell nuclei in blue. Pictures are representative of three independent experiments with three mice each. Magnification  = 200×, scale bar  = 50 µm.

In addition, Figure 3A shows that ROS production is induced *in vivo* by treatment of mouse ears with TNCB and that this production can be blocked again by pre-treatment with RF-40s or NAC.

### Source of Contact Sensitizer Induced ROS Production

To determine whether contact sensitizers induce mitochondrial ROS production, we analyzed the TNCB- or LPS- (positive control) stimulated ROS response in Pam212 and L929 cells after addition of the mitochondria specific ROS detection reagent MitoSOX™. Both stimuli induced a mitochondria-specific ROS response in both cell types (Figure 3B). Moreover, treatment of primary human keratinocytes or fibroblasts with the strong sensitizer DNCB (Figure 3C), as well as treatment of bone marrow derived DCs (BMDCs) with TNCB (Figure 4A), shows that contact allergens induce ROS also in primary cells. To assess the contribution of NADPH oxidase-induced and mitochondrial ROS to total ROS production, Pam212 and L929 cells (Figure 4B,C) were pre-treated with the mitochondria specific antioxidant ammonium pyrrolidine dithiocarbamate (APDC) [37] before stimulation with TNCB. This treatment failed to reduce the overall ROS response. Similar results were obtained when Pam212 or L929 (Figure 4B,C) cells were treated with the NADPH-oxidase specific inhibitor diphenyleneiodonium (DPI).

**Figure 6:**
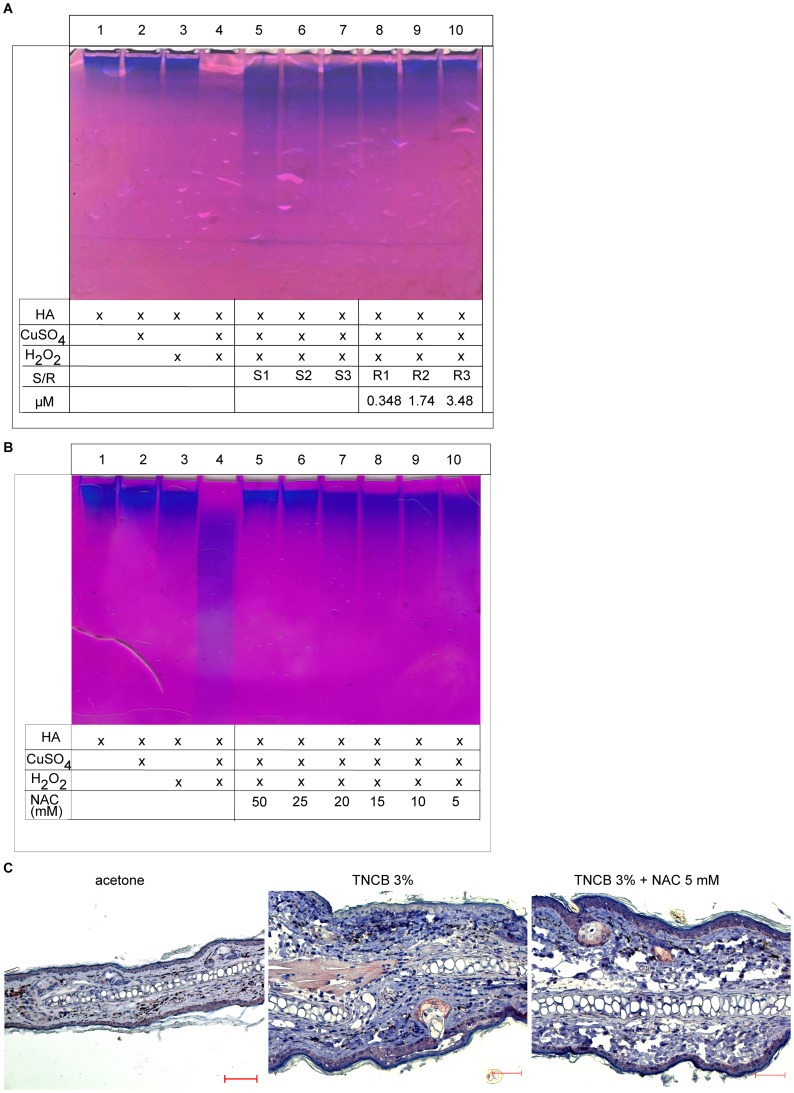
Antioxidant treatment inhibits HA degradation by ROS *in vitro* and *in vivo.* (A) High molecular weight HA was incubated with ROS inducing compounds in combination with RF-40s (R) or solvent controls (S) at corresponding concentrations. MW of HA after incubation was analyzed by SDS gel electrophoresis and staining with Stains all. The untreated HA control is shown in lane 1, CuSO_4_ or H_2_O_2_ only treated HA controls are shown in lanes 2 and 3. One representative gel out of three is shown. (B) High molecular weight HA was incubated with ROS inducing compounds as in (A) with or without addition of different concentrations of the antioxidant NAC. MW of HA after incubation was analyzed by SDS gel electrophoresis and staining with Stains all. One representative gel of three is shown. (C) Staining of HA in murine ears treated topically with acetone, TNCB or TNCB and NAC on the back side of the ear skin (upper side in panels). Samples from ears were fixed as described and paraffin sections (3 µm) were stained with bHABP with subsequent AEC (3-Amino-9-ethylcarbazole) staining and haematoxylin counterstaining. HA is stained brown/red and cell nuclei in blue. Pictures are representative of three independent experiments with three mice each. Magnification = 200×; scale bars  = 50 µm.

Recently, gp91^phox^ (Nox2) knock out mice, that lack the catalytic subunit of the superoxide-generating NADPH oxidase Nox2, have been reported to be less susceptible to dextran sulfate sodium induced colitis [38]. This effect seemed to depend on a reduced oxidative burst in the intestine. When we sensitized and challenged gp91^phox^ knock out mice with TNCB, ear swelling was normal compared to responses in wild type mice (Figure 4D), indicating that abrogating only Nox2 dependent superoxide production is not sufficient to suppress the CHS response.

Collectively, our findings demonstrate that contact sensitizers induce ROS production *in vitro* and *in vivo* that can be blocked by addition of antioxidants like RF-40s or NAC.

**Figure 7:**
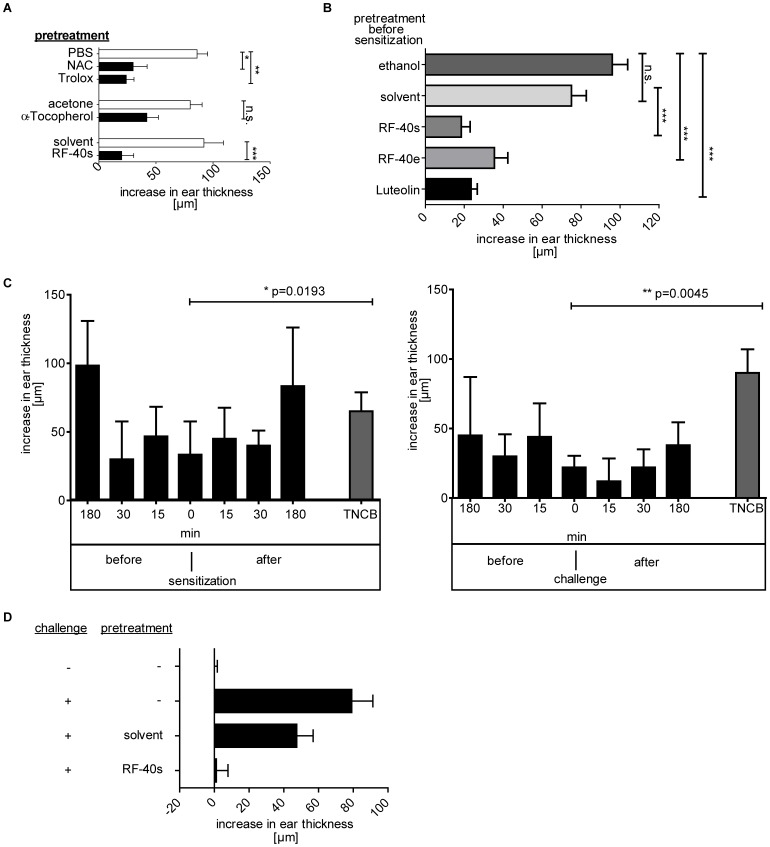
Effects of treatment with different antioxidants before and after sensitization or challenge on CHS responses. (A) Ears of mice were pre-treated by topical application of the antioxidants NAC, the hydrophilic Vitamin E analogon Trolox, the hydrophobic Vitamin Eα-Tocopherol or RF-40s or the respective solvent controls PBS, acetone or solvent before sensitization with TNCB (3%). 5 days later, increase in ear thickness was measured after challenge with TNCB (1%). Data represent the mean increase in ear thickness +/− SD of groups of five mice. One representative of two independent experiments is shown. (B) Mouse ear skin was treated 20 min before sensitization (TNCB 3%) with either RF-40s, ethanol dissolved RF-40 (RF-40e) or ethanol dissolved pure Luteolin (<98% HPLC) with the same molar concentrations of Luteolin. 24 h after challenge (TNCB 1%), increase in ear thickness was measured. The data represent the mean increase in ear thickness of groups of n = 5 mice +/− SD. One of two independent experiments is shown. (C) Ears of mice were treated with RF-40s at different times before or after sensitization (left) or challenge (right) with TNCB and increase in ear thickness was measured 24 h later. Data show mean increase in ear thickness +/− SD from one of two independent experiments with n = 5 mice/group. (D) Mice sensitized to oxazolone (3%) were either left untreated or were pre-treated with RF-40s or solvent 15 min before challenge with oxazolone (1%). Data represent mean increase in ear thickness of groups of n = 5 mice +/− SD. One representative of three independent experiments is shown.

### Immunohistochemical Analysis of HA Content in the Skin Biopsies

To investigate whether contact sensitizers are able to induce HA degradation *in vivo* we visualized the HA content in murine abdominal skin by immunohistochemistry after contact sensitizer treatment. Figure 5A shows paraffin sections obtained 2, 4, 24 or 72 h after treatment with the strong contact sensitizer TNCB. In comparison to the solvent treated controls a significant decrease in epidermal HA content was observed 24 h after contact sensitizer application. 72 h after TNCB application, re-occurrence of HA and thickening of the epidermis was observed. Untreated skin showed the same HA content as solvent treated skin (data not shown). Disappearance of epidermal HA was also observed 24 h after application of the strong sensitizer DNFB and the extreme sensitizer oxazolone (Figure 5D). Similar effects were observed when analyzing human skin treated *ex vivo* with TNCB (Figure 5B). These data suggest that contact sensitizers induce the degradation of HA in the epidermal layer of the skin to low molecular weight HA fragments that may be important mediators of the contact sensitizer induced innate inflammatory response [7], [10], [25], [39], [40], [41].

**Figure 8:**
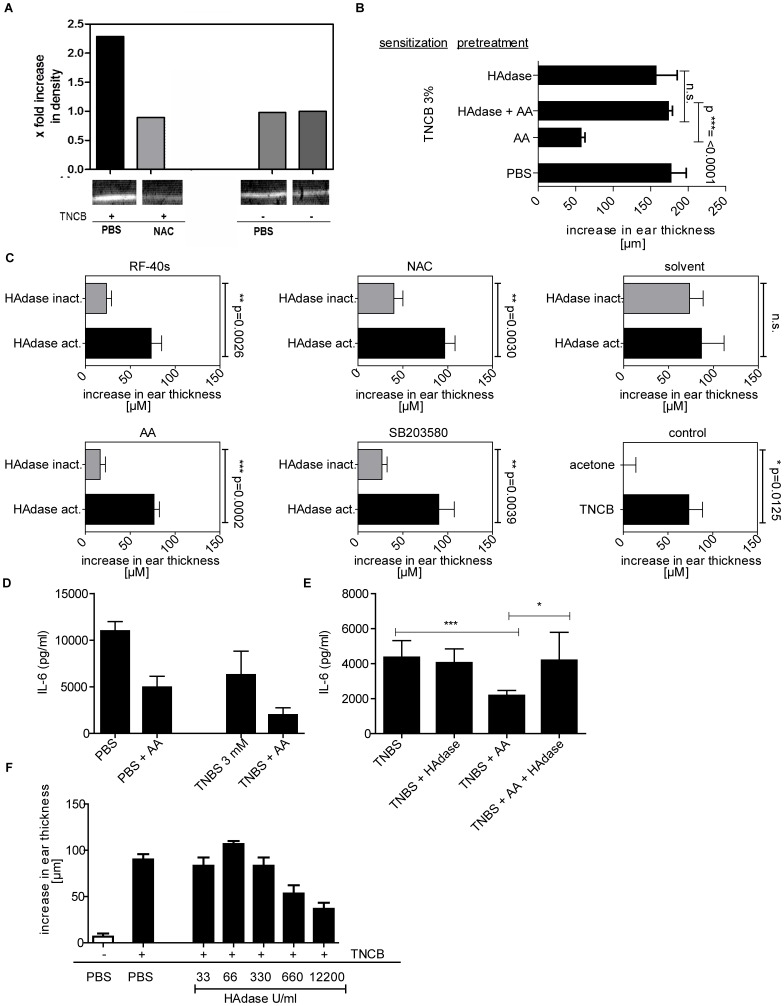
ROS induce increased hyaluronidase activity and blocking hyaluronidases prevents IL-6 production and CHS. (A) The abdomen of mice was topically pre-treated with NAC or PBS before application of TNCB (3%) for 24 h. Hyaluronidase activity was detected by hyaluronidase (HAdase) zymography and fold increase in density over untreated controls from inverted gels is shown as bars. One representative experiment of three is shown. (B) Ears of mice were pre-treated by injection of either PBS as solvent or the hyaluronidase inhibitor aristolochic acid (AA) with or without addition of hyaluronidase (660 U/ml) or hyaluronidase alone. 15 min later, mice were sensitized by topical application of TNCB on the pre-treated ears. 5 days later, ears were challenged by topical application of TNCB. Data show mean increase in ear thickness of groups of three mice +/− SD. One representative of three independent experiments is shown. (C) Ears of mice were topically pre-treated with RF-40s, solvent or NAC or by intracutaneous injection of hyaluronidase inhibitor AA or p38 MAPK inhibitor SB203580 or with acetone (solvent control for TNCB). Indicated groups were additionally injected with active or heat inactivated hyaluronidase. 15 min later, mice were sensitized. Challenged was done 5 days later as in (B). Data show mean increase in ear thickness of groups of three mice +/− SD. One representative of two independent experiments is shown. (D) Ear sheets of C57BL/6 mice were incubated with PBS in combination with AA (50 µM) or with TNBS (3 mM) and AA for 24 h. Samples were analyzed for IL-6 production by ELISA. Data show mean concentrations of IL-6+/− SD of one representative experiment out of two with 9 mice each. (E) Ear sheets of NMRI mice were incubated with TNBS (3 mM) either in combination with hyaluronidase (HAdase; 660 U/ml) or AA (50 µM) or with AA and HAdase for 24 h. Samples were analyzed for IL-6 production by ELISA. Data show mean concentrations of IL-6+/− SD of two independent experiments with n = 3 mice each. (F) Mice were pre-treated by injection of PBS or different concentrations of hyaluronidase (HAdase) into the ear pinna. Afterwards ears were treated with TNCB (3%) for sensitization and increase in ear thickness was measured 5 days later after challenge with TNCB (1%) for 24 h. Data show mean +/− SD of n = 3 mice.

To study the degradation of high MW HA to low MW fragments induced by contact sensitizers in the skin *in vivo*, we performed agarose gel electrophoresis of HA from punch biopsy samples taken from mice at different time points (untreated, 4 h and 24 h) after contact sensitizer application. Figure 5C shows the degradation of high MW HA 4 h after application of TNCB to lower MW fragments compared to the untreated control. The predominant occurrence of low MW fragments is still detectable 24 h after treatment. This confirms the ability of contact sensitizers to induce HA degradation *in vivo*.

### ROS Mediated Degradation of High MW HA can be Inhibited by Antioxidants

In order to directly assess the oxidative degradation of high MW HA and the effects of antioxidants by SDS-electrophoresis, we made use of a ROS producing Cu(II)SO_4_/H_2_O_2_ system as recently described by Gao et al. [32]. Figure 6A shows the *in chemico* degradation of high MW HA by ROS (lanes 5–7). RF40s treatment resulted in a dose dependent inhibition of degradation (lanes 8–10). In contrast, treatment of HA with combined CuSO_4_ and H_2_O_2_ (lane 4) or additionally with solvent (lane 5–6) shows the ROS mediated degradation of HA and the only weak anti-oxidative effect of solvent even at high concentrations. Similar inhibition of ROS mediated HA degradation was observed for the standard antioxidant NAC (Figure 6B). Importantly, blocking ROS production with NAC *in vivo* also inhibited HA degradation 24 h after TNCB application on ear skin as observed by HA staining in the epidermis (6C). Degradation of HA was confined to the side of the ear that was treated with TNCB (upper side in the panels). These results show the ability of ROS to directly degrade high MW HA to low MW fragments as well as the ability of both RF-40s and NAC to prevent this oxidative HA degradation.

### Antioxidant Treatment of Mouse Skin Prevents CHS Responses

Due to the pro-inflammatory role of ROS and their ability to induce HA degradation, we next analyzed the effects of topical pre-treatment with antioxidants on the development of CHS to TNCB. As shown in Figure 7A, the topical pre-treatment of the murine ear skin with NAC, Trolox or α-Tocopherol after ethanol wiping, or with RF-40s 30 min before sensitization resulted in an inhibition of the CHS response as measured by increase in ear thickness after challenge with the sensitizer. To verify that the effects of RF40s are due to its major antioxidant component, the flavonoid luteolin [34], [42], we treated mice with purified luteolin (HPLC >98%) or the respective solvent (ethanol). A similar reduction of the CHS response as seen after treatment with RF-40s was observed (Figure 7B). To further analyze the effect of RF-40s on the CHS response, we treated mice with RF-40 extract dissolved in ethanol (RF-40e) instead of the nanoparticular solvent used in RF-40s. Again, a similar reduction of CHS response compared to RF-40s or pure luteolin were observed (Figure 7B). Thus, we provide *in vivo* evidence that antioxidants are able to inhibit CHS responses to TNCB. In addition, we clearly demonstrate that the use of RF-40s is as effective as pure luteolin.

To further investigate the role of ROS in the CHS response RF-40s was applied either up to 180 min before or after sensitization or up to 180 min before or after challenge (Figure 7C). Application of RF-40s within 30 min before or after sensitization inhibited CHS responses. In addition, the application of antioxidants up to 180 min before or after challenge of sensitized mice also reduced CHS responses, indicating the potential use of antioxidants for the causative treatment of CHS responses. Notably, the inhibition of CHS was also observed in the case of the extreme sensitizer oxazolone when sensitized mice were pre-treated with RF-40s 15 min before challenge (Figure 7D). These data show that ROS crucially contribute to contact sensitizer induced skin inflammation and that blocking ROS production effectively inhibits CHS responses both in the sensitization as well as in the elicitation phase.

### Contact Sensitizers Increase Hyaluronidase Activity in Skin by a ROS Dependent Mechanism

We next investigated the ability of contact sensitizers to enhance hyaluronidase activity in the skin using hyaluronidase zymography. Figure 8A shows that extracts of skin biopsies obtained 24 h after TNCB treatment exhibited enhanced hyaluronidase activity, whereas those from controls exhibited lower activity. The increase in hyaluronidase activity after contact sensitizer treatment is ROS dependent as NAC efficiently blocks it. These results identify a role of ROS in the regulation of hyaluronidase activity and are in line with a recent study showing ROS and p38 MAPK dependent regulation of hyaluronidase 2 (Hyal 2) activity in the airway epithelium [43]. They further link contact sensitizer treatment with enhanced hyaluronidase activity levels observed in skin.

### Interference with HA Metabolism Influences CHS

To address the potential importance of the enhanced hyaluronidase activity for the CHS response we analyzed the *in vivo* effect of the hyaluronidase inhibitor aristolochic acid (AA). For this purpose AA was injected subcutaneously at non-toxic concentrations into the ear pinna of C57BL/6 mice 15 min before sensitization. Figure 8B shows that AA treatment prevented the CHS response to TNCB. To rule out that the effect of AA is due to toxicity or interference with skin penetration and to address the specificity of the AA effects we co-injected excess amount of active hyaluronidase with AA. This co-injection restored the CHS response to the level of control without AA (Figure 8B). Interestingly, injection of heat-inactivated hyaluronidase failed to do so (Figure 8C). These results show that functional hyaluronidase activity is a necessary prerequisite in the sensitization phase of CHS responses.

To further analyze the link between the contact sensitizer mediated generation of ROS and the increased hyaluronidase activity for CHS, we pre-treated ears of mice with the topically applied antioxidants NAC or RF-40s and co-injected active or heat inactivated exogenous hyaluronidase before sensitization with TNCB. Figure 8C shows the decreased CHS response in antioxidant treated compared to solvent treated mice. However, when mice were pre-treated with active hyaluronidase the inhibitory effect of the antioxidants was overcome and CHS responses were similar to untreated mice. For this effect, the enzymatic activity was crucial as heat inactivated hyaluronidase had no rescue effect for CHS in antioxidant treated mice (Figure 8C).

In order to study the potential role of p38 MAPK in the ROS mediated regulation of hyaluronidase activity mice were treated with the p38 MAPK inhibitor SB203580 before sensitization (Figure 8C). This treatment prevented the development of CHS to TNCB. Interestingly, also in this case active but not heat inactivated hyaluronidase reverted this effect indicating the involvement of p38 MAPK activation in the activation of hyaluronidases *in vivo* and, thereby, the induction of CHS responses (Figure 8C).

Preparation of ear sheets from C57BL/6 mice by mechanical separation of skin layers resulted in a trauma-induced production of the NF-κB dependent pro-inflammatory cytokine IL-6. This IL-6 production was abrogated by addition of AA to the ear sheet cultures indicating that hyaluronidase activity was required. *In vitro* treatment of the ear sheets with water-soluble 2,4,6-trinitrobenzene sulfonic acid (TNBS) resulted in reduced IL-6 production. Additional AA treatment further reduced IL-6 levels (Figure 8D). Similar effects of AA on IL-6 production were observed with ear sheets from NMRI mice treated *in vitro* with TNBS (Figure 8E). Interestingly, addition of active hyaluronidase reverted the inhibitory effects of AA. However, hyaluronidase addition to TNBS treated ear sheets did not increase IL-6 production further (Figure 8E).

In summary, these data indicate that contact sensitizer induced ROS production is crucially involved in the p38 MAPK-dependent up-regulation of hyaluronidase activity *in vivo.* Our findings demonstrate a critical role for contact sensitizer induced oxidative and enzymatic HA degradation in the induction of skin inflammation in CHS. Of note, the injection of high doses of hyaluronidase interferes with CHS (Figure 8F). This may be the result of excessive HA degradation which may prevent the timely generation of pro-inflammatory HA fragments required in the sensitization process.

## Discussion

Skin inflammation mediated by the innate immune system is a crucial step in the sensitization to contact allergens [13], [14], [27], [44]. Recent work has shown that contact sensitizers trigger innate immune mechanisms involved in anti-infectious responses [7], [18], [45], [46], [47]. These mechanisms are acting in a non-redundant collaborative manner [13], [48]. We have shown that CHS induced by organic contact sensitizers such as TNCB and oxazolone is absent in TLR2/TLR4 double deficient mice [7]. Since CHS responses develop normally in germ-free mice, we hypothesized that organic sensitizers utilize endogenous skin-derived TLR ligands to generate a pro-inflammatory tissue microenvironment [7]. This hypothesis is supported by the fact that TNCB and other organic contact sensitizers up-regulate maturation marker expression by DCs but fail to induce NF-κB dependent cytokine production by DCs *in vitro* [7]. Candidates for endogenous TLR2 and TLR4 activators are low MW HA fragments [8], [9], [10], [25], [39], [40]. High MW HA prevents TLR2 and TLR4 triggering but inflammation results in the generation of pro-inflammatory low MW HA fragments [8], [10]. Combined engagement of TLR4 and the HA receptor CD44 is needed for a full inflammatory response to HA fragments *in vitro* [40].

A putative role for HA in CHS has been described by us recently [7]. Blocking HA function with a peptide inhibitor prior to sensitization significantly reduced the CHS response to TNCB in germ-free mice. Therefore, we assumed that also in CHS high MW HA must be degraded to low MW fragments that provide necessary endogenous activators of innate inflammatory signaling in the skin.

We now show the degradation of HA in the epidermis of mice following application of contact sensitizers. This may trigger the release of soluble low MW HA and promote inflammation as described [39], [40]. Although to our knowledge no direct interaction of HA with TLR2 or TLR4 has been demonstrated so far, our data provides strong *in vivo* evidence that the degradation of HA to low molecular weight fragments is essential for the induction of a pro-inflammatory tissue micromilieu. In combination with the data that TLR2/4 expression is crucial for the induction of CHS our data therefore suggests that the inflammation caused by HA degradation involves TLR signaling. So far, we have unfortunately been unable to show an *in vitro* activation of DC using commercial low molecular weight HA fragments ranging from 2 to 12mers obtained from different suppliers, none of these fragments showed DC activating capacities (data not shown). This may be due to the fact that the exact HA fragment size required for TLR2/4 activation has not been clearly defined using synthetic material. Moreover, the activating structure *in vivo* may be different from the synthetic fragments, for example it may contain HA binding proteins as found in the ECM. Previous publications showing *in vitro* activation of DC and a role for HA in lung injury have all used their own HA preparations which make a direct comparison of the data difficult and leave open the possibility that the HA fragments from biologic material might contain either other TLR2/4 ligands or ligands for other TLRs. Biochemical data on the direct interaction of fragmented HA with TLR2/4 are urgently needed.

HA fragments can be generated either by the activity of HA degrading enzymes, the hyaluronidases, or by oxidative de-polymerization induced by ROS [31], [49], [50], [51], [52]. As HA degradation also occurs in human skin when contact sensitizers are applied *ex vivo,* our data indicate for a species spanning mechanism.

Inhibition of ROS induced HA degradation was linked to a reduction of inflammation in both bleomycin- and asbestos-induced models of pulmonary fibrosis [30], [32] as well as in a reduction of inflammatory gene expression in alveolar macrophages and epithelial cells (35). Remarkably, contact sensitizers induce ROS production both in human keratinocyte cell lines [53] as well as in DCs [54].

We demonstrate here that contact sensitizers induce ROS dependent degradation of high molecular weight HA in the skin. Our data did not show clear inhibitory effects of APDC treatment or gp91phox deficiency. This suggests a role for ROS from different cellular sources most likely including NADPH-oxidase dependent and mitochondrial ROS production. It remains to be determined whether these different ROS sources are redundant, additive or synergistic. In addition, we detect upregulation of hyaluronidase activity by contact sensitizers in the skin that promotes HA degradation. Functional inhibition of hyaluronidases by the hyaluronidase inhibitor AA abrogates not only trauma induced IL-6 production *in vitro,* but, more importantly, prevents sensitization for CHS. This inhibition is reverted when exogenous active hyaluronidase is co-administered with AA. Interestingly, inhibition of p38 MAPK activation also prevents CHS responses. This effect is at least in part dependent on the activity of hyaluronidases as co-administration of exogenous hyaluronidase reverts the inhibitory effect of the p38 MAPK inhibitor. These results are in line with a recent study showing that activation of p38 MAPK in lung inflammation results in enhanced hyaluronidase activity which in turn leads to the generation of low MW HA fragments and exaggerated inflammation [43].

So far, the functional role of HA degradation in ACD is not fully understood. In the present study, we underscore the pro-inflammatory role of HA breakdown in CHS and show that contact sensitizers modulate HA metabolism. Our data support the concept that contact sensitizer induced DAMPs serve as endogenous danger signals that are perceived by innate immune receptors [13], [48]. Thus, contact sensitizers induce HA breakdown which may result in TLR2-, TLR4- and CD44-dependent DC activation. In CHS, this HA mediated signal is delivered in the tissue microenvironment of the skin and is required for full DC activation in addition to the TLR independent induction of co-stimulatory molecules such as CD86 [7], [11]. Our data are in line with recent reports showing that congenital over-expression of hyaluronic acid synthetase 2 (HAS2) in Shar Pei dogs results in reoccurring breakdown of HA into low MW fragments, leading to the inflammatory hereditary periodic fever syndrome [55].

HA breakdown in CHS seems to be initially ROS mediated given the rapid induction of ROS by contact sensitizers. The central importance for ROS in chemical induced skin inflammation has been highlighted by the prevention of CHS involving a block of Langerhans cell migration upon keratinocyte directed over-expression of extracellular superoxide dismutase [33]. This may be due to the prevention of the oxidative and enzymatic HA degradation in the absence of sufficient amounts of ROS since ROS also regulate p38 MAPK dependent up-regulation of hyaluronidase as recently described for lung inflammation [43].

Thus, contact sensitizers induce endogenous danger signaling by triggering ROS and hyaluronidase mediated HA degradation. This process is crucial for CHS since sensitization is completely prevented by pre-treatment of the skin with antioxidants or the hyaluronidase inhibitor AA. In both cases, the inhibitory effects can be overcome by active hyaluronidase. However, at least regarding IL-6 production in ear sheets, the trauma induced IL-6 production occurring due to the mechanical separation of the ear sheets seems to result in a maximal cytokine production that can neither be significantly enhanced by addition of TNBS nor by hyaluronidase. Most interestingly, antioxidant application also prevents elicitation of CHS in sensitized animals. The role of HA induced signaling in the challenge phase of CHS remains to be determined. *In vitro* signaling studies with cells from contact allergen sensitized skin are, however, hampered by background problems due to the cell isolation procedure. Moreover, the deletion of the tissue context may significantly change cell behavior. Therefore, *in vivo* approaches using signaling inhibitors as initiated by us for p38 MAPK may provide more relevant results that could be translated into the development of novel treatment strategies.

Our findings add to an emerging, more general scheme highlighting an important functional role for ECM components as endogenous regulators of inflammation [9], [56]. Enhanced (or exaggerated) ECM degradation that disturbs the homeostasis of ECM turnover signals danger to the innate immune system. HA and biglycan are implicated in the activation of TLR2 and TLR4 signaling as well as in inflammasome activation [10], [17], [25], [57]. As shown here, blocking HA breakdown can prevent sensitization for CHS. This finding encourages the search for inhibitors of ECM degradation or for antagonists of the pro-inflammatory function of ECM components, which can be used in the prevention and therapy of inflammatory skin diseases such as ACD.

## Materials and Methods

### Ethics Statement

All of the experimental procedures were in accordance with institutional, state and federal guidelines on animal welfare. The animal experiments were approved by the Regierungspräsidium Freiburg and supervised by the Animal Protection Representatives of the University Freiburg Medical Center or the MPI.

### Mice

C57BL/6, C57BL/10 (ScSn) and NMRI mice were purchased from Charles River Laboratories or provided by the breeding facility of the Max-Planck-Institute (MPI) for Immunobiology and Epigenetics in Freiburg, Germany. TLR2/4 deficient mice [58] were also provided by the MPI. C57BL/6 gp91^phox^ mice were purchased from the Jackson Laboratories (Bar Harbor, ME) [59]. Mice were used at the age of 6–10 weeks. All of the experimental procedures were in accordance with institutional, state and federal guidelines on animal welfare. The animal experiments were approved by the Regierungspräsidium Freiburg and supervised by the Animal Protection Representatives of the University Freiburg Medical Center or the MPI.

### Media and Chemicals

The contact sensitizers 2,4,6-trinitrobenzene sulfonic acid (TNBS), 1-fluoro-2,4-dinitrobenzene (DNFB), and 4-ethoxymethylene-2-phenyloxazol-5-one (oxazolone (oxa)) were from Sigma-Aldrich, 2,4,6-trinitrochlorobenzene, (TNCB) was from VeZerf Laborsynthesen GmbH. Their classification as extreme, strong, moderate, weak is based on the Local Lymph Node assay [35], [36]. Pronase, aristolochic acid (AA), N-acetyl cysteine (NAC), ammonium pyrrolidine dithiocarbamate (APDC), diphenyleneiodonium (DPI) and the biotinylated HA binding protein (bHABP) were from Sigma-Aldrich. Hyaluronic acid (Streptococcus sp., low endotoxin) was obtained from Merck KGaA. Hyaluronic acid molecular weight markers (Select-HA™ HiLadder and LoLadder) were obtained from Amsbio. Antibody diluent with background reducing components, AEC chromogene and Dako Ultramount were from Dako Cytomation. Purified luteolin (HPLC >98%) (Carl Roth GmbH + Co. KG) was dissolved in ethanol. The *Reseda luteola* extract RF-40 (Batch 0506.06) was provided by Nahrungs-Ingenieurtechnik-GmbH (NIG) and solubilized by Aquanova AG (RF-40s) as described previously [42]. Here, “solvent” designates the nanoparticular polysorbate micelles used for the solubilization of the RF-40 extract. For a detailed description of the solubilization process and a HPLC characterization of the RF40 extract see Casetti et al. [60]. Alternatively, RF40 was solubilized in ethanol (RF40e).

### CM-H_2_DCFDA ROS Detection Assay

Determination of intracellular oxidant production was based on the oxidation of CM-H_2_DCFDA (Sigma) by intracellular ROS, resulting in the formation of the fluorescent compound 2′,7′-dichlorofluorescein (DCF). A protocol for cellular staining was adapted from Liu et al. [61]. In brief, Pam212 or L929 cells were seeded at 5×10^5^ cells/200 µl Dulbecco’s modified Eagle medium (DMEM) in black µClear 96-well plates (Greiner Bio-One). After cultivation O.N., cells were loaded with 5 µM CM-H_2_DCFDA. ROS production was induced by addition of chemicals 30 min later and determined by measuring the fluorescence of the deacetylated, oxidized DCF at OD485/528 every 10 min for 1 h using a Tekan ELISA Reader. For flow cytometric detection of ROS production in BMDC, cells were seeded at 2×10^5^ cells per well into 96 well plates. Cells were pre-treated by addition of 2.5 mM NAC or equivalent volumes of PBS (solvent control) for 15 min. After addition of contact sensitizer, cells were incubated for 1.5 h at 37°C, 5% CO_2_ before addition of 5 µM CM-H_2_DCFDA and further incubation for 30 min. After washing with PBS, DCF fluorescence was analyzed using a FACSCanto^TM^II (BD).

### Dihydroethidium (DHE) ROS Detection Assay

Ears of mice were topically treated with antioxidants and respective solvents either 15 min (RF-40s) or 1 h and in addition 15 min (NAC) before topical application of TNCB. Before NAC or PBS application, ears were wiped excessively with ethanol to allow penetration of NAC. 15 min after TNCB application, ears were taken after euthanasia and incubated *ex vivo* for 30 min with 5 mM DHE (Sigma) in DMSO. 8 mm punches were applied to glass slides and ROS production was monitored using fluorescence microscopy as oxidation of DHE results in the generation of the fluorescent 2-hydroxyethidium.

### Detection of Mitochondrial ROS Production

Pam212 or L929 cells were seeded at 4×10^5^ cells per well in 200 µl DMEM in 8-well Lab-Tek II glass chamber slides (Nalgene Nunc). After cultivation overnight, cells were stimulated for 1 h with chemicals and subsequently loaded with 200 µl MitoSOX™ (Invitrogen) (5 mM Stock in ethanol dissolved in HBSS to working solution of 5 µM) for 10 min. After three washing steps with HBSS, cells were fixed in ice cold acetone for 2 s, nuclei were counterstained with DAPI and slides were covered with Fluoromount (Dako Cytomation) before detection of mitochondrial ROS formation as red staining by fluorescence microscopy.

### Immunohistochemistry

Skin samples were fixed in HA-fixative as described by Lin et al [62]. Paraffin sections (3 µm) were fixed on slides overnight at 60°C and deparaffinized by Rotihistol (Carl Roth) treatment. After antigen demasking in citrate buffer in a steamer at 100°C for 30 min, permeabilisation was carried out with PBS/0.5% Tween 20 for 15 min at RT. After washing in TBS, endogenous peroxidase activity was blocked by incubation with 0.3% H_2_O_2_ for 10 min at 4°C. Following three washing steps with TBS for 5 min each, unspecific receptor binding was blocked by incubation for 1 h with TBS/1% BSA at RT. The sections were then incubated overnight at 4°C with bHABP (25 µg/ml) diluted in antibody diluent with background reducing agents (Dako).

Detection of the bHABP was carried out using the DCS Chromoline Diagnostic System (DCS Innovative Diagnostik-Systeme). In brief, slides were incubated for 20 min with streptavidin conjugated horse-radish peroxidase (1∶20) (HRP-Label, DCS), washed three times with TBS for 5 min and incubated at RT with 3-amino-9-ethylcarbazole (AEC) substrate solution until distinct red coloring was observed. After washing with dH_2_O for 2 min, sections were counterstained with haematoxylin and mounted with Dako Ultramount.

### Detection of ROS Induced HA Degradation *in vitro*

ROS were generated by the Cu(II)/H_2_O_2_ system as published [32]. In brief, both superoxide anion and hydroxyl radical generation was induced by incubation of 0.1 M NaH_2_PO_4_ (Merck) pH 7.4 containing 50 µM CuSO_4_ (Sigma-Aldrich) with 100 µM H_2_O_2_ (Carl Roth). Different concentrations of RF-40s or solvent were added to 20 µg high molecular weight hyaluronic acid (Calbiochem) and ROS inducing agents for 1 h at room temperature. Samples were analyzed by electrophoresis through a 7% polyacrylamide gel and visualized by overnight staining with 0.005% Stains-All dye (Sigma-Aldrich) in 50% ethanol. Bromophenol blue served as tracking marker for sample movement.

### Induction of Contact Hypersensitivity (CHS)

CHS was induced as described [7]. In brief, mice were sensitized by application of 150 µl Oxazolone or 100 µl of TNCB (both 3% w/v unless otherwise indicated) or respective volumes of solvent control on the shaved abdomen of 3–5 mice/group. Ear thickness was measured on day 5 and mice were challenged by application of 20 µl contact sensitizer (1%) or respective solvent control to the ears. 24 h later, the increase in ear thickness was measured.

### Influence of Antioxidants, Hyaluronidase- or p38 MAPK Inhibitors on CHS Responses

Ear skin of mice was pre-treated by topical application of 20 µl NAC (5 mM) or RF40s (5.24 µM) or respective solvents 30 min before sensitization with 20 µl 3% TNCB. Alternatively, antioxidant treatment was done in sensitized mice on day 5 at different time points as indicated. Hyaluronidase inhibitor aristolochic acid (AA, 20 µl, 50 µM) or p38 MAPK inhibitor (SB203580, 20 µl, 0.02 µM) was injected into the ear pinna 15 min before sensitization with 20 µl TNCB (3%) on the back side of the ears. Mock sensitization with acetone served as control. Five days later, mice were challenged by application of 20 µl TNCB (1%) on the back side of contralateral ears. Increase in ear thickness was measured 24 h later with a thickness gauge (Mitutoyo).

Non-toxic AA concentrations were determined by LDH assay *ex vivo* on ear sheet culture samples and live/dead fluorescence microscopy (data not shown). Some treatment groups were co-injected with hyaluronidase (660 U/ml, active or heat inactivated (5 min, 99°C)) to assess the reversion of the inhibitory effects of antioxidants, the p38 MAPK inhibitor or AA on the CHS response. This treatment also revealed the non-toxic effects of AA *in vivo*.

### Zymography Assays

Ears of mice were treated with chemicals for 24 h. Incubation of 8 mm punch biopsies was done in lysis buffer (50 mM Tris pH8.0, 1 mM EDTA, 150 mM NaCl_2_, 0.5% Na-desoxycholate, 1% Nonidet P40). Zymographic detection of hyaluronidase activity was carried out as described [63]. In brief, samples were electrophoresed through 10% SDS-polyacrylamide gels containing 0.17 mg/ml of high MW HA. After electrophoresis, gels were washed in 50 mM HEPES buffer pH 7.4 containing 3% Triton X-100 and subsequently incubated in 0.15 M NaCl_2_/0.1 M Na-formate, pH 3.5 at 37°C for 18 h. Gels were then stained with 0.5% Alcian blue in 3% acetic acid where hyaluronidase activity was visualized as clear bands on the blue background. For the assessment of relative activity, intensities of the bands were recorded using a Epson Biostep View Pix 700 with SilverFast-SE software (LaserSoft Imaging AG,) and analyzed using Quantity One software (Bio-Rad).

### Measurement of IL-6 Production in Ear Sheet Cultures

Ears of mice were separated in dorsal and ventral halves using forceps. Culture of the ear sheets was carried out in 6 well plates with 1.5 ml RPMI +10% FCS/well in an incubator at 37°C and 5% CO_2_. IL-6 production was analyzed 24 h after treatment with TNBS, AA and hyaluronidase or PBS as control as indicated using a commercial IL-6 ELISA kit (OptEia-Kit, Becton Dickinson) according to manufacturer instructions and a Tekan ELISA Reader.

### Analysis of Molecular Size of HA in Skin Samples by Agarose Gel Electrophoresis

Detection of the molecular size of HA samples derived from abdominal skin either untreated or treated with TNCB (3%) for 4 h or 24 h was carried out according to the protocol by Lee et al. [64]. In brief, 10 mm punch biopsies of skin samples were digested for 16 h at 55°C in Pronase (5 U/10 mg sample) in a total volume of 200 µl according to a protocol by Wang et al. [65] before inactivation of Pronase by heating to 100°C for 10 min. Afterwards, samples were mixed with loading buffer (2 M sucrose +0.02% bromophenolblue) and electrophoresed through a 0.5% agarose gel in TAE buffer for 8 h at a constant current of 2.5 V/cm (50 V). HA was visualized by staining in 0.005% Stains all (Fluka) overnight in the dark and destaining under normal light in water until distinct bands were visible.

### Statistics

Data were analysed using the unpaired Student t -test (two-tailed), and statistical significance was established at P≤0.05.Data are expressed as mean ± SD if not indicated otherwise.

## References

[1] Martin SF (2004). T lymphocyte-mediated immune responses to chemical haptens and metal ions: implications for allergic and autoimmune disease.. Int Arch Allergy Immunol.

[2] Vocanson M, Hennino A, Rozieres A, Poyet G, Nicolas JF (2009). Effector and regulatory mechanisms in allergic contact dermatitis.. Allergy.

[3] Saint-Mezard P, Berard F, Dubois B, Kaiserlian D, Nicolas JF (2004). The role of CD4+ and CD8+ T cells in contact hypersensitivity and allergic contact dermatitis.. Eur J Dermatol.

[4] Martin SF, Dudda JC, Delattre V, Bachtanian E, Leicht C (2004). Fas-mediated inhibition of CD4+ T cell priming results in dominance of type 1 CD8+ T cells in the immune response to the contact sensitizer trinitrophenyl.. J Immunol.

[5] Kimber I, Dearman RJ (2002). Allergic contact dermatitis: the cellular effectors.. Contact Dermatitis.

[6] Jeong E, Lee JY (2011). Intrinsic and extrinsic regulation of innate immune receptors.. Yonsei Med J.

[7] Martin SF, Dudda JC, Bachtanian E, Lembo A, Liller S (2008). Toll-like receptor and IL-12 signaling control susceptibility to contact hypersensitivity.. J Exp Med.

[8] Jiang D, Liang J, Fan J, Yu S, Chen S (2005). Regulation of lung injury and repair by Toll-like receptors and hyaluronan.. Nat Med.

[9] Powell JD, Horton MR (2005). Threat matrix: low-molecular-weight hyaluronan (HA) as a danger signal.. Immunol Res.

[10] Scheibner KA, Lutz MA, Boodoo S, Fenton MJ, Powell JD (2006). Hyaluronan fragments act as an endogenous danger signal by engaging TLR2.. J Immunol.

[11] Tesar BM, Jiang D, Liang J, Palmer SM, Noble PW (2006). The role of hyaluronan degradation products as innate alloimmune agonists.. Am J Transplant.

[12] Lutz MB, Schuler G (2002). Immature, semi-mature and fully mature dendritic cells: which signals induce tolerance or immunity?.

[13] Martin SF, Esser PR, Weber FC, Jakob T, Freudenberg MA (2011). Mechanisms of chemical-induced innate immunity in allergic contact dermatitis.. Allergy.

[14] Kaplan DH, Igyarto BZ, Gaspari AA (2011). Early immune events in the induction of allergic contact dermatitis.. Nat Rev Immunol.

[15] Seong SY, Matzinger P (2004). Hydrophobicity: an ancient damage-associated molecular pattern that initiates innate immune responses.. Nat Rev Immunol.

[16] Schaefer L, Babelova A, Kiss E, Hausser HJ, Baliova M (2005). The matrix component biglycan is proinflammatory and signals through Toll-like receptors 4 and 2 in macrophages.. J Clin Invest.

[17] Babelova A, Moreth K, Tsalastra-Greul W, Zeng-Brouwers J, Eickelberg O (2009). Biglycan, a danger signal that activates the NLRP3 inflammasome via toll-like and P2X receptors.. J Biol Chem.

[18] Weber FC, Esser PR, Muller T, Ganesan J, Pellegatti P (2010). Lack of the purinergic receptor P2X(7) results in resistance to contact hypersensitivity.. J Exp Med.

[19] Kogan G, Soltes L, Stern R, Gemeiner P (2007). Hyaluronic acid: a natural biopolymer with a broad range of biomedical and industrial applications.. Biotechnol Lett.

[20] Nusgens BV (2010). [Hyaluronic acid and extracellular matrix: a primitive molecule?].. Ann Dermatol Venereol.

[21] Delmage JM, Powars DR, Jaynes PK, Allerton SE (1986). The selective suppression of immunogenicity by hyaluronic acid.. Ann Clin Lab Sci.

[22] Campo GM, Avenoso A, Campo S, D’Ascola A, Nastasi G (2010). Molecular size hyaluronan differently modulates toll-like receptor-4 in LPS-induced inflammation in mouse chondrocytes.. Biochimie.

[23] Campo GM, Avenoso A, Campo S, D’Ascola A, Traina P (2010). Differential effect of molecular mass hyaluronan on lipopolysaccharide-induced damage in chondrocytes.. Innate Immun.

[24] Noble PW (2002). Hyaluronan and its catabolic products in tissue injury and repair.. Matrix Biol.

[25] Termeer C, Benedix F, Sleeman J, Fieber C, Voith U (2002). Oligosaccharides of Hyaluronan activate dendritic cells via toll-like receptor 4.. J Exp Med.

[26] Garantziotis S, Li Z, Potts EN, Kimata K, Zhuo L (2009). Hyaluronan mediates ozone-induced airway hyperresponsiveness in mice.. J Biol Chem.

[27] Jiang D, Liang J, Noble PW (2011). Hyaluronan as an immune regulator in human diseases.. Physiol Rev.

[28] Schmidt M, Raghavan B, Muller V, Vogl T, Fejer G (2010). Crucial role for human Toll-like receptor 4 in the development of contact allergy to nickel.. Nat Immunol.

[29] Martin SF (2010). T cell recognition of chemicals, protein allergens and drugs: towards the development of in vitro assays.. Cell Mol Life Sci.

[30] Eberlein M, Scheibner KA, Black KE, Collins SL, Chan-Li Y (2008). Anti-oxidant inhibition of hyaluronan fragment-induced inflammatory gene expression.. J Inflamm (Lond).

[31] Agren UM, Tammi RH, Tammi MI (1997). Reactive oxygen species contribute to epidermal hyaluronan catabolism in human skin organ culture.. Free Radic Biol Med.

[32] Gao F, Koenitzer JR, Tobolewski JM, Jiang D, Liang J (2008). Extracellular superoxide dismutase inhibits inflammation by preventing oxidative fragmentation of hyaluronan.. J Biol Chem.

[33] Na K, Kim KE, Park ST, Kim TY (2007). EC-SOD suppresses contact hypersensitivity in mouse skin by impairing Langerhans cell migration.. J Invest Dermatol.

[34] Woelfle U, Simon-Haarhaus B, Merfort I, Schempp CM (2010). Reseda luteola L. extract displays antiproliferative and pro-apoptotic activities that are related to its major flavonoids.. Phytother Res.

[35] Gerberick GF, Ryan CA, Kern PS, Schlatter H, Dearman RJ (2005). Compilation of historical local lymph node data for evaluation of skin sensitization alternative methods.. Dermatitis.

[36] Kern PS, Gerberick GF, Ryan CA, Kimber I, Aptula A (2010). Local lymph node data for the evaluation of skin sensitization alternatives: a second compilation.. Dermatitis.

[37] Zhou R, Yazdi AS, Menu P, Tschopp J (2011). A role for mitochondria in NLRP3 inflammasome activation.. Nature.

[38] Bao S, Carr E, Xu YH, Hunt NH (2011). Gp91(phox) contributes to the development of experimental inflammatory bowel disease.. Immunol Cell Biol.

[39] Taylor KR, Trowbridge JM, Rudisill JA, Termeer CC, Simon JC (2004). Hyaluronan fragments stimulate endothelial recognition of injury through TLR4.. J Biol Chem.

[40] Taylor KR, Yamasaki K, Radek KA, Di Nardo A, Goodarzi H (2007). Recognition of hyaluronan released in sterile injury involves a unique receptor complex dependent on Toll-like receptor 4, CD44, and MD-2.. J Biol Chem.

[41] Termeer CC, Hennies J, Voith U, Ahrens T, Weiss JM (2000). Oligosaccharides of hyaluronan are potent activators of dendritic cells.. J Immunol.

[42] Woelfle U, Esser PR, Simon-Haarhaus B, Martin SF, Lademann J (2011). UVB-induced DNA damage, generation of reactive oxygen species, and inflammation are effectively attenuated by the flavonoid luteolin in vitro and in vivo.. Free Radic Biol Med.

[43] Monzon ME, Fregien N, Schmid N, Falcon NS, Campos M (2010). Reactive oxygen species and hyaluronidase 2 regulate airway epithelial hyaluronan fragmentation.. J Biol Chem.

[44] Martin SF, Jakob T (2008). From innate to adaptive immune responses in contact hypersensitivity.. Curr Opin Allergy Clin Immunol.

[45] Sutterwala FS, Ogura Y, Szczepanik M, Lara-Tejero M, Lichtenberger GS (2006). Critical role for NALP3/CIAS1/Cryopyrin in innate and adaptive immunity through its regulation of caspase-1.. Immunity.

[46] Watanabe H, Gaide O, Petrilli V, Martinon F, Contassot E (2007). Activation of the IL-1beta-processing inflammasome is involved in contact hypersensitivity.. J Invest Dermatol.

[47] Klekotka PA, Yang L, Yokoyama WM (2010). Contrasting roles of the IL-1 and IL-18 receptors in MyD88-dependent contact hypersensitivity.. J Invest Dermatol.

[48] Freudenberg MA, Esser PR, Jakob T, Galanos C, Martin SF (2009). Innate and adaptive immune responses in contact dermatitis: analogy with infections.. G Ital Dermatol Venereol.

[49] Manzanares D, Monzon ME, Savani RC, Salathe M (2007). Apical oxidative hyaluronan degradation stimulates airway ciliary beating via RHAMM and RON.. Am J Respir Cell Mol Biol.

[50] Yamazaki K, Fukuda K, Matsukawa M, Hara F, Matsushita T (2003). Cyclic tensile stretch loaded on bovine chondrocytes causes depolymerization of hyaluronan: involvement of reactive oxygen species.. Arthritis Rheum.

[51] Casalino-Matsuda SM, Monzon ME, Conner GE, Salathe M, Forteza RM (2004). Role of hyaluronan and reactive oxygen species in tissue kallikrein-mediated epidermal growth factor receptor activation in human airways.. J Biol Chem.

[52] Casalino-Matsuda SM, Monzon ME, Forteza RM (2006). Epidermal growth factor receptor activation by epidermal growth factor mediates oxidant-induced goblet cell metaplasia in human airway epithelium.. Am J Respir Cell Mol Biol.

[53] Mehrotra P, Mishra KP, Raman G, Banerjee G (2005). Differential regulation of free radicals (reactive oxygen and nitrogen species) by contact allergens and irritants in human keratinocyte cell line.. Toxicol Mech Methods.

[54] Mizuashi M, Ohtani T, Nakagawa S, Aiba S (2005). Redox imbalance induced by contact sensitizers triggers the maturation of dendritic cells.. J Invest Dermatol.

[55] Docampo MJ, Zanna G, Fondevila D, Cabrera J, Lopez-Iglesias C (2011). Increased HAS2-driven hyaluronic acid synthesis in shar-pei dogs with hereditary cutaneous hyaluronosis (mucinosis).. Vet Dermatol.

[56] Sorokin L (2011). The impact of the extracellular matrix on inflammation.. Nat Rev Immunol.

[57] Yamasaki K, Muto J, Taylor KR, Cogen AL, Audish D (2009). NLRP3/Cryopyrin Is Necessary for Interleukin-1{beta} (IL-1{beta}) Release in Response to Hyaluronan, an Endogenous Trigger of Inflammation in Response to Injury.. J Biol Chem.

[58] Lembo A, Kalis C, Kirschning CJ, Mitolo V, Jirillo E (2003). Differential contribution of Toll-like receptors 4 and 2 to the cytokine response to Salmonella enterica serovar Typhimurium and Staphylococcus aureus in mice.. Infect Immun.

[59] Pollock JD, Williams DA, Gifford MA, Li LL, Du X (1995). Mouse model of X-linked chronic granulomatous disease, an inherited defect in phagocyte superoxide production.. Nat Genet.

[60] Casetti F, Jung W, Wolfle U, Reuter J, Neumann K (2009). Topical application of solubilized Reseda luteola extract reduces ultraviolet B-induced inflammation in vivo.. J Photochem Photobiol B.

[61] Liu A, Arbiser JL, Holmgren A, Klein G, Klein E (2005). PSK and Trx80 inhibit B-cell growth in EBV-infected cord blood mononuclear cells through T cells activated by the monocyte products IL-15 and IL-12.. Blood.

[62] Lin W, Shuster S, Maibach HI, Stern R (1997). Patterns of Hyaluronan Staining Are Modified by Fixation Techniques.. J Histochem Cytochem.

[63] Guntenhoner MW, Pogrel MA, Stern R (1992). A substrate-gel assay for hyaluronidase activity.. Matrix.

[64] Lee HG, Cowman MK (1994). An agarose gel electrophoretic method for analysis of hyaluronan molecular weight distribution.. Anal Biochem.

[65] Wang Q, Teder P, Judd NP, Noble PW, Doerschuk CM (2002). CD44 deficiency leads to enhanced neutrophil migration and lung injury in Escherichia coli pneumonia in mice.. Am J Pathol.

